# The roles of DGAT1 and DGAT2 in human myotubes are dependent on donor patho‐physiological background

**DOI:** 10.1096/fj.202300960RR

**Published:** 2023-10-01

**Authors:** Zehra Irshad, Jenny Lund, Anne Sillars, Nils Gunnar Løvsletten, Seley Gharanei, Ian P. Salt, Dilys J. Freeman, Jason M. R. Gill, G. Hege Thoresen, Arild C. Rustan, Victor A. Zammit

**Affiliations:** ^1^ Translational and Experimental Medicine, Warwick Medical School University of Warwick Coventry UK; ^2^ Section for Pharmacology and Pharmaceutical Biosciences, Department of Pharmacy University of Oslo Oslo Norway; ^3^ School of Cardiovascular and Metabolic Health, College of Medical, Veterinary and Life Sciences University of Glasgow Glasgow UK; ^4^ Warwickshire Institute for the Study of Diabetes, Endocrinology and Metabolism (WISDEM) University Hospitals Coventry and Warwickshire NHS Trust Coventry UK; ^5^ School of Molecular Biosciences, College of Medical, Veterinary and Life Sciences University of Glasgow Glasgow UK; ^6^ Department of Pharmacology, Institute of Clinical Medicine University of Oslo Oslo Norway

**Keywords:** diacylglycerol acyltransferases, fatty acid oxidation, insulin resistance, lipogenesis, muscle lipid, triacylglycerols

## Abstract

The roles of DGAT1 and DGAT2 in lipid metabolism and insulin responsiveness of human skeletal muscle were studied using cryosections and myotubes prepared from muscle biopsies from control, athlete, and impaired glucose regulation (IGR) cohorts of men. The previously observed increases in intramuscular triacylglycerol (IMTG) in athletes and IGR were shown to be related to an increase in lipid droplet (LD) area in type I fibers in athletes but, conversely, in type II fibers in IGR subjects. Specific inhibition of both diacylglycerol acyltransferase (DGAT) 1 and 2 decreased fatty acid (FA) uptake by myotubes, whereas only DGAT2 inhibition also decreased fatty acid oxidation. Fatty acid uptake in myotubes was negatively correlated with the lactate thresholds of the respective donors. DGAT2 inhibition lowered acetate uptake and oxidation in myotubes from all cohorts whereas DGAT1 inhibition had no effect. A positive correlation between acetate oxidation in myotubes and resting metabolic rate (RMR) from fatty acid oxidation in vivo was observed. Myotubes from athletes and IGR had higher rates of de novo lipogenesis from acetate that were normalized by DGAT2 inhibition. Moreover, DGAT2 inhibition in myotubes also resulted in increased insulin‐induced Akt phosphorylation. The differential effects of DGAT1 and DGAT2 inhibition suggest that the specialized role of DGAT2 in esterifying nascent diacylglycerols and de novo synthesized FA is associated with synthesis of a pool of triacylglycerol, which upon hydrolysis results in effectors that promote mitochondrial fatty acid oxidation but decrease insulin signaling in skeletal muscle cells.

## Graphical abstract

DGAT1 and DGAT2 exert opposing effects on muscle cell insulin sensitivity through changes in precursors/products of the respective pools of TAG they synthesize.

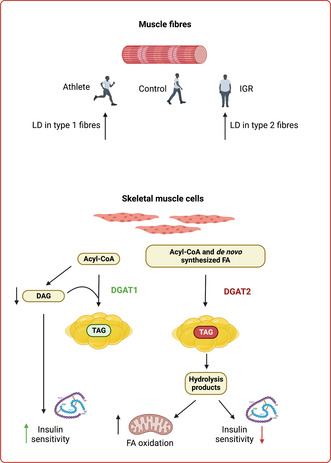

**Abbreviations**
ASM: acid‐soluble metabolites
DAG: diacylglycerol
DGAT: diacylglycerolacyltransferase
HG: high glucose concentration
IGT: impaired glucose tolerance
IMTG: intramyocellular triacylglycerol
LD: lipid droplets
NG: normal glucose concentration
OGTT: oral glucose tolerance test
T2DM: type 2 diabetes mellitus
TAG: triacyglycerol

## 1 INTRODUCTION

Ectopic triacylglycerol (TAG) accumulation in skeletal muscle (intramyocellular triglyceride, IMTG) is observed both in conditions characterized by peripheral insulin resistance (IR), such as obesity and type 2 diabetes mellitus (T2DM), and in the highly insulin‐sensitive muscle of exercise‐trained individuals.^1^, ^2^ Because of this association of similar overall levels of IMTG with extremes of insulin sensitivity, an extensive literature has emerged on the possibility that pools of metabolically related complex lipids—acyl‐CoA, diacylglycerols, ceramide^3^, ^4^—that affect insulin sensitivity are heterogeneous both in terms of composition and intracellular compartmentation.^5^, ^6^ In particular, the relationships between different DAG species—in terms of their structure and acyl residue composition—and insulin sensitivity and mitochondrial function of muscle have been extensively studied.^5^, ^7^ These studies have not shown any definitive relationships between overall DAG content of muscle fibers and the decreased insulin sensitivity that would have been expected from in vitro studies showing that 1,2‐DAG activates atypical protein kinase C isoforms that attenuate insulin action. Indeed, most of the data show paradoxical increases in DAG species in the more insulin‐sensitive muscles of exercise‐trained individuals.^8^, ^9^ These data have led to suggestions that the compartmentation of DAG in muscle fibers is critical for the etiology of insulin resistance in muscle.^7^, ^9^, ^10^ Detailed analyses of the isomers of muscle DAG have not resolved this paradox suggesting that an alternative mechanism may link high overall DAG levels with insulin resistance in muscle.

A potentially important determinant of the multiplicity of DAG species formed in muscle and other tissues after lipolysis of IMTG is the route used for the *synthesis* of TAG in the first instance due to the existence of two distinct diacylglycerol acyltransferases (DGAT1 and DGAT2), which between them account for the vast majority of the final reaction involved in TAG synthesis. The two DGATs are co‐expressed in many cell types but whereas DGAT1 is exclusively anchored within the endoplasmic reticulum, DGAT2 can additionally be located within lipid droplets (LDs) or associated with mitochondria.^11^, ^12^ When assayed in vitro, DGAT1 and DGAT2 activities have overlapping specificities for sn‐1,3 and sn‐2,3 DAGs^13^ and are considerably redundant in their ability to esterify preformed long‐chain exogenous fatty acids (FA).^14^ However, DGAT2 is specialized for the synthesis of TAG from nascent DAG—that is, those formed through the glycerol‐3‐phosphate (G3P) pathway—and de novo synthesized FA. This has been demonstrated in hepatocytes,^15^ brown adipocytes,^16^ and human skeletal muscle‐derived myotubes.^17^ In primary brown adipocytes, the considerable flux of glucose into TAG is entirely dependent on DGAT2 activity and proceeds concomitantly with hydrolysis of the newly formed TAG.^16^

This specialization of DGAT2 suggests that the TAG in LDs synthesized by this isoform may give rise to hydrolysis products different from those generated by hydrolysis of TAG synthesized by DGAT1. It is well‐established for several cell types that not only do exogenous pre‐formed FA have to be converted to TAG before they can be oxidized,^18^ but also that signaling by fatty acids is dependent on their first being esterified to TAG, followed by subsequent hydrolysis of that TAG to generate modulators of transcription factors that may affect signal transduction and mitochondrial function.^19^, ^20^, ^21^ In this context, it is noteworthy that a role for the use of de novo synthesized FA in the generation of insulin resistance‐mediating lipids has been described previously.^22^ Although no increased de novo DAG synthesis was detected in muscles of obese and T2DM individuals,^6^ previous in vitro studies on human skeletal muscle‐derived myotubes demonstrated that hyperglycemic conditions increase rates of de novo lipogenesis through activation of SREBP1c.^23^

The respective roles of DGAT1 and DGAT2 in skeletal muscle have previously been addressed through experiments involving the overexpression of the respective genes in mice. Overexpression of DGAT1 in heart and muscles resulted in increased TAG accumulation but improved insulin sensitivity, an effect ascribed to the increased clearance of acyl‐CoA and DAG from the relevant metabolic compartments.^24^, ^25^ Conversely, overexpression of DGAT2 in type II muscle fibers exacerbated muscle IR in vivo^26^ suggesting that TAG formed via the DGAT2 route generates lipolytic products that promote IR. These studies had the limitation of mediating overexpression of the two DGATs in different cell types but they suggest that TAG synthesized by DGAT1 may lead to distinct compartmentation (e.g., in distinct LDs) and signaling from that synthesized through DGAT2.

We have previously shown, through the use of selective inhibitors of DGAT1 and DGAT2, that cultured human myotubes provide a valid model for studying metabolic adaptations induced by pathophysiological changes in the donors.^17^ Glucose and FA metabolism in human muscle‐derived myotubes retain many of the phenotypic differences observed in vivo.^27^, ^28^ Therefore, in this study, we have used myotubes cultured from individuals with impaired glucose regulation (IGR), endurance‐trained individuals (athletes), and lean controls to test whether the route of TAG synthesis via DGAT1 vs. DGAT2 affects metabolism of exogenous oleic acid and of the lipogenic substrate acetate, and the association between substrate uptake/oxidation, insulin sensitivity of myotubes, and relevant physiological parameters of the respective donors.

## 2 MATERIALS AND METHODS

### 2.1 Materials

Dulbecco's modified Eagle's medium (DMEM)‐Glutamax low glucose with sodium pyruvate, Dulbecco's phosphate‐buffered saline (DPBS) without Mg^2+^ and Ca^2+^, defatted fetal bovine serum (FBS), penicillin–streptomycin (10 000 IE/mL), and amphotericin B were from Thermo Fisher Scientific (Waltham, MA, US). 96‐well Corning CellBIND tissue culture plates were from Corning (Schiphol‐Rijk, the Netherlands). Ultroser G was from Pall Life Sciences (Cergy‐Saint‐Christophe, France). Insulin (Actrapid) was from Novo Nordisk (Bagsvaerd, Denmark). Bovine serum albumin (BSA) essentially FA‐free, L‐carnitine, D‐glucose, oleic acid (18:1, n‐9), HEPES, DMSO, gentamicin, and the DGAT1 inhibitor, T863, were from Sigma‐Aldrich (St. Louis, MO, US). The DGAT2 inhibitor, JNJ‐DGAT2‐A, was from Janssen Research and Development (High Wycombe, UK). UniFilter‐96 GF/B microplates, Isoplate‐96 scintillation microplates, TopSeal‐A transparent film, and Ultima Gold scintillation fluid were from PerkinElmer (Shelton, CT, US). Bio‐Rad Protein Assay Dye Reagent Concentrate was from Bio‐Rad (Copenhagen, Denmark). [1‐^14^C]oleic acid (56–59 mCi/mmol) and [1‐^14^C]acetate (50.5 mCi/mmol) were from PerkinElmer NEN (Boston, MA, US).

### 2.2 Methods

#### 2.2.1 Ethics statement

Muscle biopsies were obtained after written informed consent. The study was conducted with ethical approval from the West of Scotland Research Ethics Committee 4 (reference: 16/WS/0002) and was conducted according to the principles outlined in the Declaration of Helsinki.

#### 2.2.2 Inclusion and exclusion criteria for recruitment of male subjects

The study aimed to recruit three distinct groups of phenotypically different white European men aged 30–60 years (Table 1). These were a group (*n* = 11) of lean, healthy, and physically inactive men termed “control”; a group (*n* = 13) of endurance‐trained athletes termed “athlete” and a group of men (*n* = 12), who were diagnosed with either impaired fasting glucose (IFG) (fasting glucose between 6.1 and 6.9 mM), impaired glucose tolerance (IGT) (plasma glucose ≥7.8 and ≤11.1 mM following a 2‐h oral glucose tolerance test [OGTT]) or had T2DM (fasting plasma glucose level ≥7.0 mM, glucose ≥11.1 mM post 2 h oral OGTT or HbA1c >48 mmol/mol) but were treatment naïve, collectively termed “IGR” (impaired glucose regulation).

**TABLE 1:** Donor anthropometric characteristics and performance parameters.

	Athlete (*n* = 13)	Control (*n* = 11)	IGR (*n* = 12)	Athlete vs Control	IGR vs Control	Athlete vs IGR
				*p*‐value		
Age (years)	42.8 ± 2.0	49.3 ± 2.6	53.0 ± 1.6	.06	.23	**<.001**
Weight (kg)	74.7 ± 1.8	77.1 ± 1.5	96.9 ± 3.6	.33	**<.001**	**<.001**
BMI (kg/m^2^)	23.1 ± 0.4	24.1 ± 0.6	30.8 ± 1.1	.18	**<.001**	**<.001**
Percentage body fat (%)	11.6 ± 1.7	19.9 ± 1.8	32.4 ± 2.4	**<.001**	**<0.001**	**<.001**
Lean mass (Kg)	64.2 ± 5.3	61.0 ± 5.9	64.8 ± 6.4	.37	.33	.92
Fasting blood glucose (mM)	5.2 ± 0.5	5.2 ± 0.5	8.1 ± 1.6	.38	**<.001**	**<.001**
Plasma Insulin (μU/mL)	4.4 ± 2.0	5.9 ± 1.7	15.3 ± 13.7	.85	**<.001**	**<.001**
HbA1c (%)	5.3 ± 0.1	5.2 ± 0.1	7.0 ± 0.2	.29	**<.001**	**<.001**
Insulin sensitivity index	10.6 ± 1.1	6.6 ± 0.4	2.3 ± 0.4	**<.001**	**<.001**	**<.001**
HOMA‐IR	1.0 ± 0.2	1.3 ± 0.1	6.9 ± 2.0	.12	**.02**	**<.001**
VO_2max_ (mL.kg^−1^.min^−1^)	55.3 ± 1.7	34.3 ± 1.6	30.8 ± 2.1 (*n* = 10)	**<.001**	.20	**<.001**
Lactate threshold (%)	65.1 ± 1.2	49.2 ± 1.5	51.4 ± 1.6 (*n* = 10)	**<.001**	.31	**<.001**
RMR kcal.day^−1^	1686 ± 147	1597 ± 162	1894 ± 252	.32	.47	.08
RMR Fat (%)	41.2 ± 6.1 (*n* = 12)	50.5 ± 3.7	48.9 ± 6.1	.21	.82	.38

*Note*: Data are given as means ± SEM. Comparison between groups was by unpaired Student's *t*‐test. Lactate threshold given as % of VO_2max_. RMR fat %, proportion of whole body resting energy expenditure coming from fat oxidation. *p* values indicating statistical significance are shown in bold.

Abbreviations: BMI, body mass index; HbA1c, glycated hemoglobin; HOMA‐IR, homeostatic model assessment of insulin resistance; IGR, impaired glucose regulation; VO_2max_, maximal oxygen uptake.

Exclusion criteria for the whole study were female sex; age <30 years or >60 years; ethnicity other than white European; uncontrolled hypertension defined as systolic blood pressure >160 mmHg or diastolic blood pressure >90 mmHg; previous history of coronary heart disease or stroke; and a serious chronic health condition.

Inclusion criteria depended on the specific cohort. For the control group, they were body mass index (BMI) 18–27 kg/m^2^; HbA1c < 43.8 mmol/mol (<6%); not engaging in regular vigorous physical activity (less than one hour per week); no history of cardiovascular, metabolic, or other systemic disease; and no medication for lipid or carbohydrate metabolic disorders. Inclusion criteria for the athlete group were BMI 18–27 kg/m^2^; HbA1c < 43.8 mmol/mol (<6%); undertaking >5 h per week of vigorous endurance‐based exercise for ≥2 years; no history of cardiovascular, metabolic, or of other systemic disease; no medication for lipid or carbohydrate metabolic disorders. Inclusion criteria for the IGR group were diagnosis of IFG or IGT, or a diagnosis of T2DM controlled by lifestyle changes only.

#### 2.2.3 Metabolic and physiological measurements

Potential participants were screened using the tests below to ensure adherence to the inclusion criteria. Each individual completed an International Physical Activity Questionnaire (IPAQ, Geneva 2002). Participants who met the criteria were required to attend two further visits that were spaced by at least 7 days. For the first visit, participants were asked to fast overnight and to abstain from vigorous activity for the prior 48 h. During this visit, participants first had anthropometric measurements taken, including height, weight, and percentage body fat assessed by air displacement plethysmography (BodPod, Life Measurement Instruments, California, USA). Following this, they underwent an OGTT in the morning and then, in the afternoon, performed a maximal incremental cycle ergometer test (Lode B.V., Groningen, the Netherlands) with work rate increasing, from a starting load of 50 W, by 15 W/min (control and IGR groups) or 25 W/min (athletes) until volitional fatigue. Expired air was collected throughout using breath‐by‐breath cardiopulmonary exercise testing (CPET) machine (Quark, COSMED Srl, Middlesex, UK) to measure oxygen uptake (VO_2_) and carbon dioxide production (VCO_2_). Achievement of maximal oxygen uptake (VO_2max_) was verified by volitional exhaustion together with a rate of perceived exertion (RPE) of 19–20, a respiratory exchange ratio >1.15, and heart rate within 10 beats of age‐predicted maximum. Lactate threshold was calculated noninvasively as the point where the gradient in the VCO_2_/VO_2_ relationship started to increase from the initial linear relationship using the V‐slope method.^29^

During the OGTT, venous blood samples were taken in the fasted state and at 30 min intervals for 120 min following a 75 g oral glucose. Glucose was measured in 0.5 mL plasma samples obtained from blood collected in EDTA (0.1 mM final concentration) using a YSI2300 Stat Analyzer (YSI Corp. Ohio, US). HbA1c was measured using a reverse‐phase cation exchange HPLC using a Menarinni HA‐8180 Analyzer (Meranini Diagnostics Srl, Florence, Italy). The insulin sensitivity index (ISI) was calculated as described in Ref.^30^ The Homeostasis Assessment Model (HOMA) was used to calculate insulin resistance (HOMA IR), first described in Ref.^31^ Resting energy expenditure (REE) was measured using the QUARK Resting Metabolic Rate (RMR) metabolic cart (COSMED Srl, Middlesex, UK). This system consists of a canopy hood with plastic skirting, turbine flow meter with sampling line, 3 L calibration syringe, antibacterial filter, and OMNIA PC software loaded onto a PC. REE measurements were made first thing in the morning, with the participants in the fasted state, and having been asked to refrain from vigorous physical activity for 48‐h prior to the visit. Then, a muscle biopsy was taken from the *vastus lateralis* muscle on the left leg under local anesthesia (lidocaine hydrochloride) using an automatic biopsy needle (Magnum Biopsy System, Bard, Covington, GA, US). The biopsy was divided and used for different analyses. Myoblasts were prepared from satellite cells obtained from approximately 100 mg of *vastus lateralis* muscle and were frozen and stored in liquid nitrogen prior to activation and proliferation to myotubes.

#### 2.2.4 Histological staining

Cryosections of *vastus lateralis* muscle samples (7 μm) were co‐stained for lipid (Bodipy 493/503–green) and for anti‐human myosin heavy chain (hMYH)7 to stain type I fibers largely as described previously.^32^ Briefly, after fixation in formalin, sections were washed 3 times in PBS and blocked using 10% goat serum. After washing with PBS, they were incubated with anti‐hMYH7, followed by dye‐labeled secondary antibodies (red) to visualize the type I fibers. They were further stained with Bodipy 493/503 (green) in the dark. They were then washed in PBS, dried, covered with Prolong Gold™, and mounted.

#### 2.2.5 Culture of myotubes

Multinucleated myotubes were established by activation and proliferation of myoblasts obtained from satellite cells. The cells were proliferated to myoblasts that were stored in liquid nitrogen until used for further culturing as previously described.^33^ The cells were cultured on multiwell plates in DMEM‐Glutamax (5.5 mM glucose) supplemented with 2% FBS, 25 IU penicillin, 25 μg/mL streptomycin, 1.25 μg/mL amphotericin B, 50 ng/mL gentamicin, and 2% Ultroser G. When they reached approximately 80% confluency, the growth medium was replaced by DMEM‐Glutamax (5.5 mM glucose) supplemented with 2% FBS, 25 IU penicillin, 25 μg/mL streptomycin, 1.25 μg/mL amphotericin B, 50 ng/mL gentamicin, and 25 pM insulin. During culturing and differentiation, the cells were incubated under a humidified atmosphere (5% CO_2_) at 37°C, and the medium was changed every 2–3 days. Experiments were performed on myotubes after 6–7 days of differentiation.

#### 2.2.6 Substrate oxidation assay and acid‐soluble metabolites (ASM)

Myotubes were cultured in 96‐well CellBIND microplates. Once fully differentiated, cells were pretreated for 6 h with DMEM‐Glutamax, either in 5.5 mM glucose (normoglycemia, NG) or 20 mM glucose (hyperglycemia, HG). Some wells were supplemented with 10 μM DGAT1 inhibitor (D1i) or 10 μM DGAT2 inhibitor (D2i) with or without the presence of 100 nM insulin (only for HG conditions). Thereafter, the media were changed to substrate oxidation medium consisting of DPBS w/HEPES (10 mM) supplemented with either 100 μM (0.5 μCi/mL) [1‐^14^C]oleic acid or 100 μM (2 μCi/mL) [1‐^14^C]acetate, L‐carnitine, BSA, 0.1% DMSO, with or without 10 μM D1i or 10 μM D2i. A 96‐well UniFilter microplate, activated for the capture of CO_2_ by the addition of 1 M NaOH, was mounted on top of the tissue culture plate. After incubation for 4 h, the cells were washed twice with PBS and harvested in 0.1 M NaOH. The ^14^CO_2_ trapped in the filter and cell‐associated (CA) substrate was measured with a 2450 MicroBeta^2^ scintillation counter (PerkinElmer).

Incomplete FA β‐oxidation was assessed by measuring acid‐soluble metabolites (ASM) produced from [1‐^14^C]oleic acid (0.5 μCi/mL, 100 μM) released by the myotubes using the method described in.^34^ Briefly, 100 μL radiolabelled incubation medium from the substrate oxidation assay was transferred to a tube and acidified with 300 μL ice‐cold perchloric acid (1 M); 30 μL BSA (6%) was added as carrier. The precipitated protein was pelleted by centrifugation at 2000 × *g* for 10 min at 4°C. An aliquot (200 μL) of the supernatant was transferred to a scintillation vial and 3 mL of Ultima Gold scintillation fluid was added and radioactivity quantified by liquid scintillation (Packard Tri‐Carb 1900 TR, PerkinElmer).

The sum of ^14^C‐ASM, ^14^CO_2_, and cell‐associated (CA) radioactivity was considered to represent the total cellular uptake of oleic acid (ASM + CO_2_ + CA). All data were normalized to total cell protein content in the cell lysates, using the Bio‐Rad protein assay.

#### 2.2.7 Measurement of de novo lipogenesis from acetate

Once fully differentiated, cells were incubated for 6 h with DMEM‐Glutamax, either in 5.5 mM glucose (NG) or 20 mM glucose (HG). Selected wells were supplemented with 10 μM D1i or 10 μM D2i with or without 100 nM insulin (only for HG conditions). Myotubes were then incubated in DPBS w/HEPES with 100 μM (2 μCi/mL) [1‐^14^C]acetate for 4 h and cell lysates were prepared by adding 0.1 M NaOH. Total lipids were isolated by filtration of the lysates through hydrophobic MultiScreen HTS plates (Millipore, Billerica, MA, US) and the radioactivity associated with different classes of lipids was determined by scintillation counting. Rates of lipogenesis from acetate were calculated and normalized to lysate protein content.

#### 2.2.8 Quantification of mRNA expression

Muscle biopsies (approximately 50 mg) were initially homogenized in 1 mL of TRIzol® Reagent (Invitrogen) and total RNA isolated according to the manufacturer's instructions. Potential DNA contamination was removed via DNA‐free™ DNase treatment (Ambion) as per manufacturer's instructions. cDNA was reverse transcribed from RNA using a High Capacity cDNA Reverse Transcriptase Kit (Applied Biosystems, Warrington, UK) according to manufacturer's instructions. Target gene expression was quantitated relative to a control gene (*RPL19* Hs02338565_gH) using commercial primer probe sets (Thermo Fisher Scientific: *ACACA* Hs01046047_m1, *ACACB* Hs01565914_m1, *CD36* Hs00354519_m1, *DGAT1* HS01017541_m1, *DGAT2* Hs01045913_m1, *GLUT4* Hs00168966_m1, *IL6* HS00174131_m1, *MYH2* Hs00430042_m1, *MYH7* Hs01110632_m1, *PLIN5* Hs00965990_m1, *PPARA* Hs00947536_m1, *PPARGC1A* Hs00173304_m1, *SCD* Hs01682761_m1, *SREBF1* Hs01088691_m1, *TNF* Hs00174128_m1) in a final volume of 25 μL on a StepOnePlus Real‐Time PCR system (Thermo Fisher Scientific) using the protocol of 50°C for 2 min, 95°C for 10 min, then 40× of 95°C for 15 s and 60°C for 1 min, according to the manufacturer's instructions. Quantification analysis was carried out using StepOnePlus v2.3 software which calculated the threshold cycle (C_T_) values. For mRNA quantification in whole‐muscle samples, the expression of target assays was normalized by subtracting the C_T_ value of the endogenous control (*hL19*) from the C_T_ value of the target assay. The fold increase relative to the control was calculated using the 2^−ΔCT^. The expression of the target assay was then expressed as a percentage relative to the endogenous control assay. The sequences of the respective primers used are given in Data S1.

Differentiated myotubes were incubated for 6 h in DMEM medium containing either 5.5 mM glucose (NG) or 20 mM glucose (HG) both in the presence or absence of combinations of 100 nM insulin, 10 μM D1i or 10 μM D2i. Total RNA was extracted using TRIzol reagent. cDNA was prepared using reverse transcription. Briefly, RNA (1 μg) was mixed with oligodT (1 μL) in a final volume of 12 μL by adding RNase free water. Samples were heated at 70°C for 5 min before chilling on ice. Subsequently, 8 μL of mixture containing RNase inhibitor (10 U/μl), dNTPs (10 mM), Bioscript reverse transcriptase, and RNase free water were added to each sample. Samples were heated at 40°C for 60 min and the reaction was stopped by heating to 70°C for 10 min. The cDNA formed was mixed with 180 μL of nuclease‐free water and stored at −20°C. Samples were thawed only once before quantification. RT‐PCR was performed using SYBR green dye and expression for all the samples (*n* ≥ 3) was calculated by using the ΔCt method, factoring in the efficiencies of each primer. For mRNA quantification in myotubes, the variances of input cDNA were normalized against levels of hL19 as the housekeeping gene. Melting curve analysis confirmed amplification specificity.

#### 2.2.9 Quantification of Akt(Ser473) phosphorylation by immunoblotting

Differentiated myotubes were incubated for 6 h in DMEM media containing either 5.5 mM (NG) or 20 mM (HG) glucose in the presence or absence of D1i and/or D2i, both at 10 μM. This concentration was chosen in light of previous dose‐inhibition studies performed, which showed that at this concentration approximately 70% of DGAT1 or DGAT2 activities were inhibited without loss of cell viability.^15^, ^16^, ^17^ Cells were further incubated in the absence or presence of 10 nM insulin for 10 min. Myotubes were scraped into lysis buffer (50 mM Tris–HCl pH 7.4 at 4°C, 150 mM NaCl, 50 mM NaF, 5 mM Na pyrophosphate, 1 mM Na orthovanadate, 1 mM EDTA, 1 mM EGTA, 1 mM dithiothreitol, 0.1 mM benzamidine, 0.1 mM phenylmethylsulfonyl fluoride, 5 mg/L soybean trypsin inhibitor, 1% [v/v] Triton X‐100, 1% [v/v] glycerol) on ice. Lysates were centrifuged and supernatants stored at −20°C. Protein concentration of myotube lysates was determined using the Bradford method and equal amounts of protein lysate were resolved by SDS‐PAGE and then immunoblotted with rabbit anti‐phospho‐Akt Ser473 (Cell Signaling Technologies, cat. no. 4058) or mouse anti‐Akt antibodies (Cell Signaling Technologies, cat. no. 2920) diluted 1:500 in TBS‐T (50 mM Tris, 150 mM NaCl, 0.1% [v/v] Tween‐20) and 3% (w/v) BSA. Differentiated myotubes were incubated for 6 h in DMEM media containing either 5.5 mM (NG) or 20 mM (HG) glucose in the presence or absence of D1i and/or D2i, both at 10 μM. Cells were further incubated in the absence or presence of 10 nM insulin for 10 min. Myotubes were scraped into lysis buffer (50 mM Tris–HCl pH 7.4 at 4°C, 150 mM NaCl, 50 mM NaF, 5 mM Na pyrophosphate, 1 mM Na orthovanadate, 1 mM EDTA, 1 mM EGTA, 1 mM dithiothreitol, 0.1 mM benzamidine, 0.1 mM phenylmethylsulfonyl fluoride, 5 mg/L soybean trypsin inhibitor, 1% [v/v] Triton X‐100, 1% [v/v] glycerol) on ice. Lysates were centrifuged and supernatants stored at −20°C. Protein concentration of myotube lysates was determined using the Bradford method and equal amounts of protein lysate were resolved by SDS‐PAGE and then immunoblotted with rabbit anti‐phospho‐Akt Ser473 (Cell Signaling Technologies, cat. no. 4058) or mouse anti‐Akt antibodies (Cell Signaling Technologies, cat. no. 2920) diluted 1:500 in TBS‐T (50 mM Tris, 150 mM NaCl, 0.1% [v/v] Tween‐20) and 3% (w/v) BSA. Proteins were visualized using infrared dye‐labeled secondary antibodies on a LI‐COR Odyssey infrared imaging system or horseradish preoxidase‐labeled secondary antibodies by enhanced chemiluminescence. The two methods gave equivalent results which were pooled. Densitometric quantification of band intensity (phospho‐Akt relative to total Akt) was determined using ImageJ gel analysis software.

#### 2.2.10 Lipid droplet counting and sizing

Cryosections (7 μm) of muscle biopsies fixed and costained for type I fibers (red) using cognate anti‐myosin antibodies and for lipid, using Bodipy 493/503 (green) were used. Distinction between LD in type I and type II fibers was performed by counting LD numbers only in the green channel using Image J software on the open‐source Fiji platform.^35^ The number of LD per unit area was first quantified when the red channel was attenuated (i.e., when the entire field was green) to quantify the LD numbers in both type I and type II fibers. The population of LD specifically in type II fibers was then quantified in the green areas when the red channel was turned on. The difference between these two values represents the number of LD per unit area of type I fibers (stained red). In addition, the software was used to assign LD diameters to ten intervals in increments intervals of 0.010 μm. The data were used to calculate the overall cross‐sectional area of lipid per unit cell surface area (LD‐CSA) by multiplying the number of LDs in each diameter size range by the nominal cross‐sectional area of a spherical droplet for the median diameter in that range.

#### 2.2.11 Presentation of data and statistical analyses

Data are presented as means ± SEM for *n* numbers of individual muscle donors, either as absolute values or as a percentage of the respective controls, as appropriate. Each experimental determination was performed at least in duplicate. Where indicated, statistical analyses were performed using Student's *t*‐test (GraphPad version 9.3.1) and by linear mixed‐model analysis in SPSS version 29 (IBM SPSS Statistics for Macintosh, Armonk, NY, US). Linear mixed‐model analysis was used to compare differences between conditions with within‐donor variation and simultaneous comparison of differences between groups with between‐donor variation. Using the linear mixed‐model analysis enabled the inclusion of all observations even when they were not independent. Statistical interactions between the analyzed groups were tested in SPSS. A *p* value ≤.05 was considered significant. Nonparametric correlations were performed with Spearman's rho (two‐tailed) test and are presented as correlation coefficient (*r*). A *p* value **≤**.05 was considered significant.

## 3 RESULTS

### 3.1 Donor characteristics and performance parameters

Demographics of the participants in each of the three groups are presented in Table 1. The IGR group had a significantly higher age than the athlete group, and a higher BMI than the control and athlete groups, while the athlete group had lower percentage body fat than both control and IGR groups. The athlete group had a substantially higher maximal oxygen uptake (VO_2max_) than the other two groups, and VO_2max_ in the control group was higher than the IGR group. In addition, lactate threshold was higher for athletes compared to both control and IGR subjects. As expected, the IGR group had higher mean HbA1c levels than the control and athlete groups, and the mean IGR HbA1c level was within the diagnostic range for T2DM (>48 mmol/mol). Mean values for HOMA‐IR were markedly higher in the IGR group than either the control or the athlete groups. ISI was in the order: athletes > control > IGR.

### 3.2 Lipid droplet density and size distribution in type I and type II muscle fibers

**FIGURE 1:**
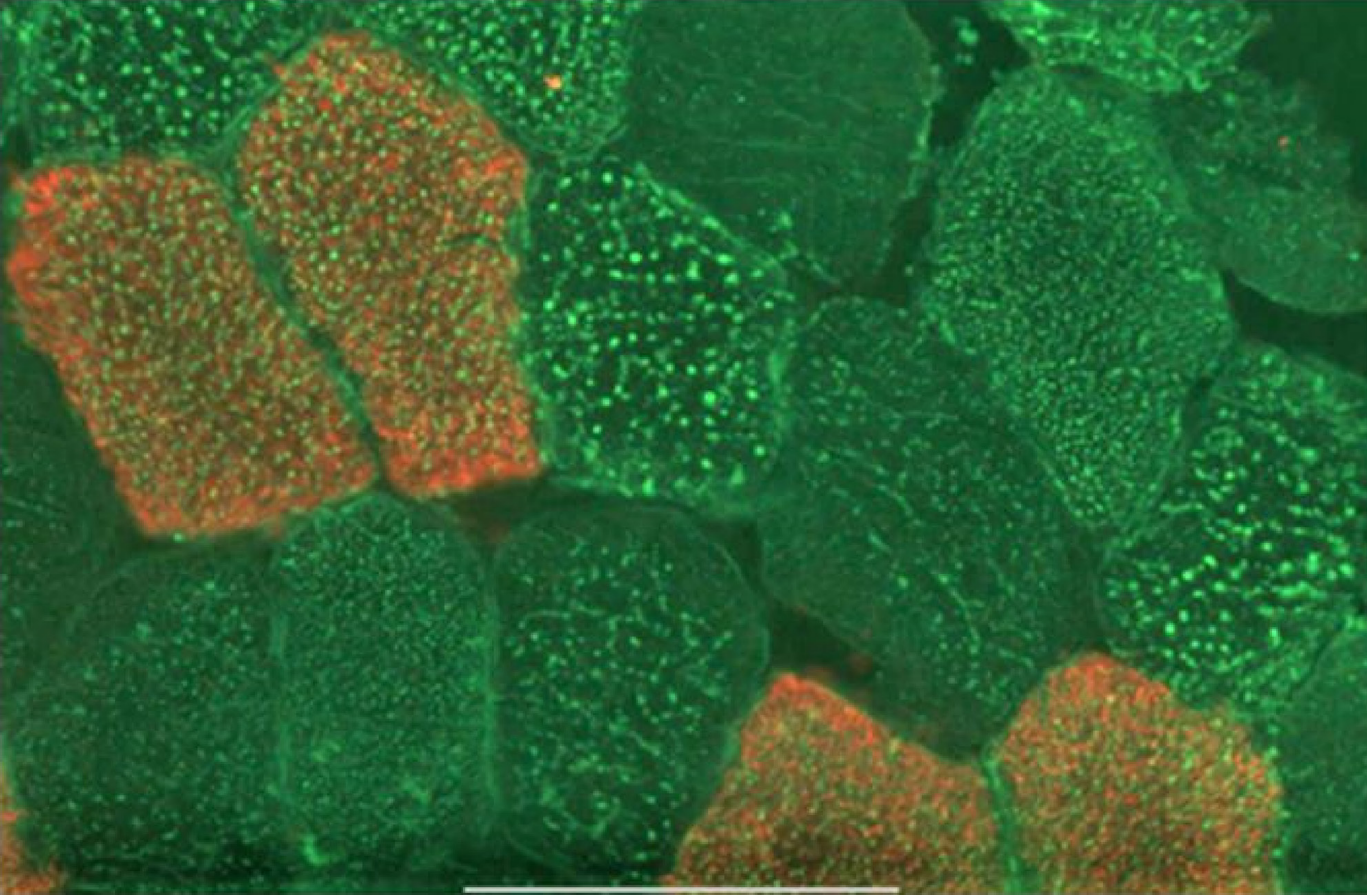
A representative light micrograph of a cryosection from a *vastus lateralis* muscle biopsy obtained from an IGR individual. After fixation in formalin, sections were washed 3 times in PBS and blocked using 10% goat serum. They were then incubated with anti‐hMYH7, followed by dye‐labeled secondary antibodies (red) to visualize the type I fibers. They were further stained with Bodipy 493/503 (green) in the dark. They were then washed in PBS, dried, covered with Prolong Gold™, and mounted. The bar indicates 100 μm.

**FIGURE 2:**
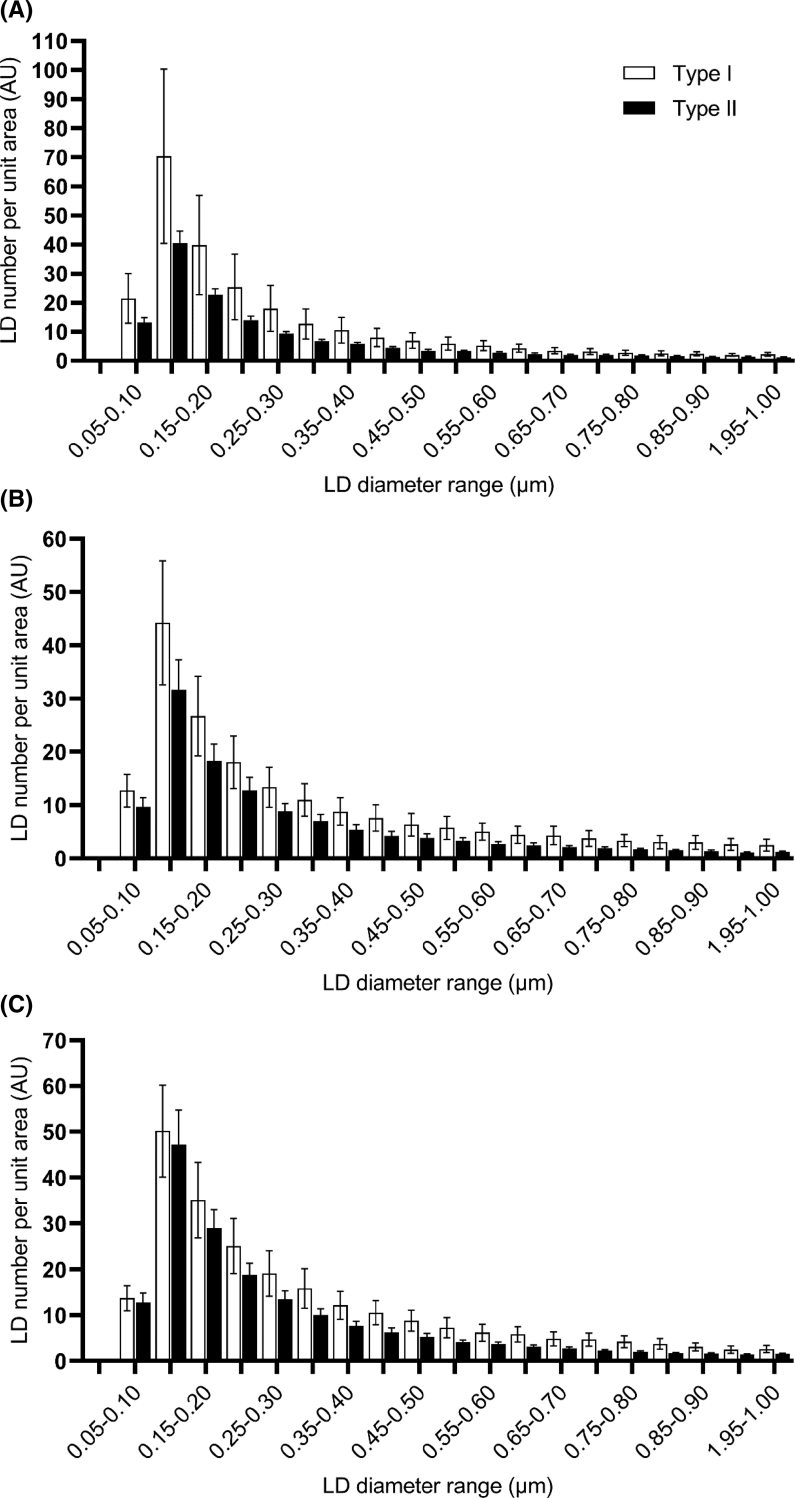
The size distribution of lipid droplets (LD) in sections of *vastus lateralis* muscle biopsies obtained from (A) athlete, (B) control and (C) impaired glucose regulation (IGR) cohorts in type I (white bars) and type II muscle fibers. Cryosections were obtained and stained for lipid and type I myosin and LD numbers per unit cell area in the different size ranges indicated were performed as described in the Methods section. Athlete (*n* = 7), control (*n* = 8) and IGR (*n* = 7).

The limited amount of material obtained in each biopsy precluded the quantification of the total amount of lipid per muscle fiber. But it allowed us to determine the number and size of LDs per cross‐sectional area in type I and type II fibers. The differential staining of the fiber types (see Figure 1) was used solely as a method to enable counting of LDs per unit area in each type. In all three cohorts the size distribution of LD was monophasic and peaked within the 0.10 to 0.15 μm range for both fiber types (Figure 2). However, there were differences in the relative number of droplets in the different size ranges in type I and type II fibers between cohorts. In biopsies from all three cohorts, there was a higher LD number per unit area in type I than in type II fibers, as expected from the higher oxidative capacity of the former. However, in the lower ranges of LD diameters in sections obtained from IGR subjects, there were no such differences between fiber types due to an increase in LD number in type II fibers of diameters lower than 0.15 μm. This difference in LD number per unit area was most pronounced in athletes (Figure 2A). These data were reflected in the overall cross‐sectional area of lipid per unit cell surface area (LD‐CSA) computed by multiplying the number of LDs in each diameter size range by the nominal cross‐sectional area of a spherical droplet for the median diameter in that range. Therefore, LD‐CSA corresponds to area under the curve for the plots presented in Figure 2. The data in Table 2 show that there was significantly higher LD‐CSA in type I compared to type II fibers only in sections from athletes. The higher lipid content of type I fibers evident for the control and IGR cohorts did not reach statistical significance. Whereas the LD‐CSA was similar for type I fibers for all three cohorts, the LD‐CSA for type II fibers was significantly higher for the IGR cohort compared to controls and athletes. Therefore, the previously established increase in the lipid content of muscle in IGR^1^, ^2^ appears to be associated primarily with type II fibers, whereas the increase in lipid content in athletes muscle occurred predominantly in type I fibers.

**TABLE 2:** Lipid droplet cross‐sectional area in type I and type II fibers per unit cell area (LD‐CSA) in sections of biopsies obtained from muscles of individuals from athlete, control and IGR cohorts.

	Type I fibers LD‐CSA per unit CA (AU)	Type II fibers LD‐CSA per unit CA (AU)
Athletes (*n* = 7)	25.1 ± 1.2^#^	12.6 ± 1.5
Control (*n* = 8)	21.3 ± 8.2	11.6 ± 1.8
IGR (*n* = 7)	26.0 ± 7.4	15.1 ± 1.8*

*Note*: LD‐CSA values were calculated as the summation of the product of the multiplication of the number of lipid droplets of different sizes by the area computed for each diameter in the ranges shown in Figure 1. Values are expressed in arbitrary units (AU) and are means ± SEM for the number of individuals sampled. The total LD‐CSA is given together with the CSA for LD or three diameter ranges, namely, <0.2, 0.2–0.6 and >0.6 μm. Statistical evaluation was by Student's *t*‐test.

Abbreviations: CA, cell area; IGR, impaired glucose regulation.

^#^ Significantly different between type I and type II fibers for athletes (*p* < .05).

* Significantly different between type II fibers in IGR vs controls and athletes (*p* < .05).

### 3.3 Expression of mRNA in whole muscle biopsies

**FIGURE 3:**
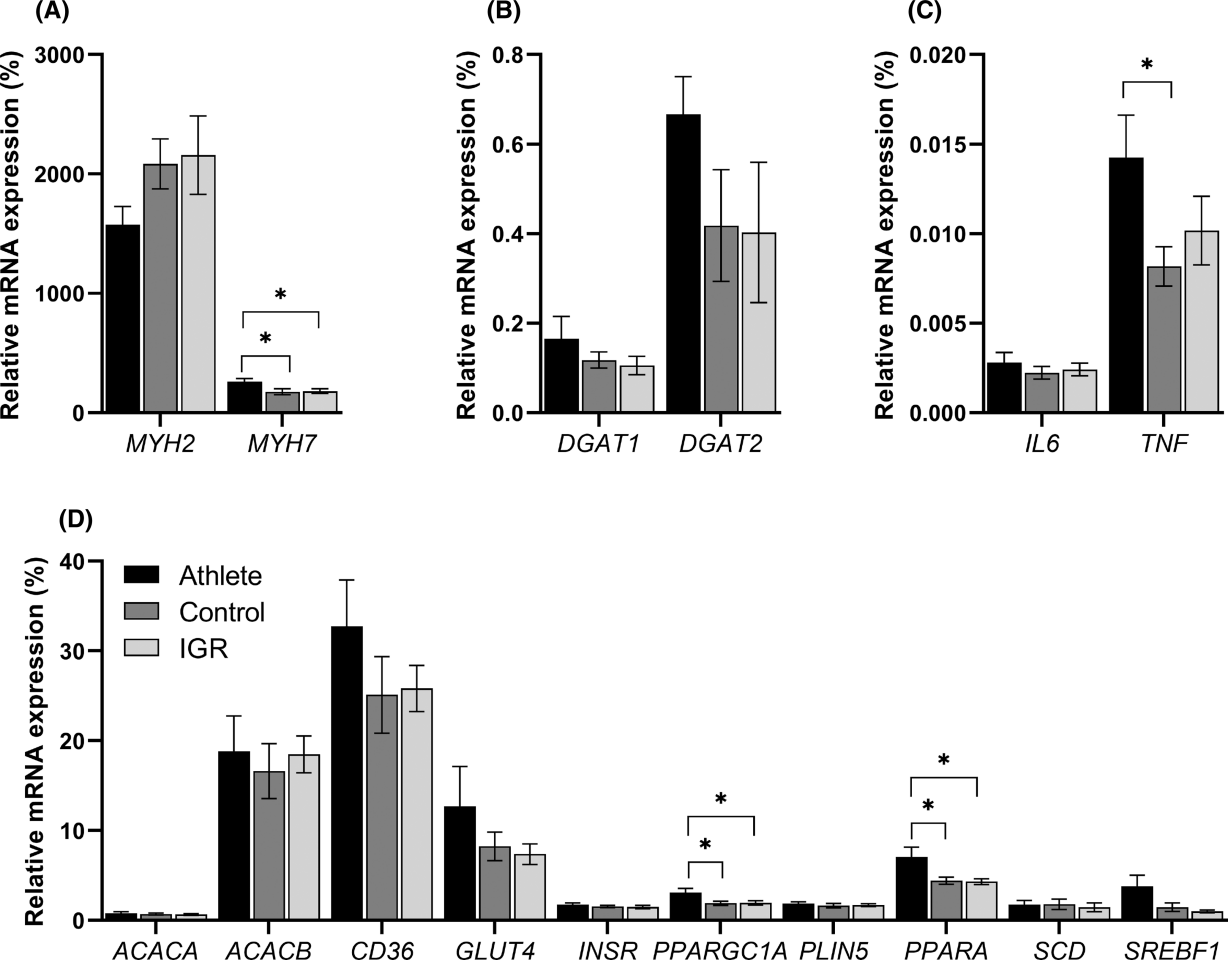
Expression of mRNA in whole *vastus lateralis* muscle biopsies. mRNA expression was measured in biopsy samples from the *vastus lateralis* muscle obtained from men in the three cohorts studied. Values were normalized with respect to the expression of *hL19* mRNA as housekeeping (*HK*) gene. Athletes (*n* = 11), control (*n* = 10) and IGR (*n* = 10). (A) mRNA expression of myosin heavy chain 2 and 7 (*MYH2* and *MYH7*); (B) diacylglycerol acyltransferase 1 and 2 (*DGAT1* and *DGAT2*); (C) Interleukin 6 (*IL6*) and tumor necrosis factor (*TNF*); (D) acetyl‐CoA carboxylase 1 (*ACACA*), acetyl‐CoA carboxylase 2 (*ACACB*), CD36 molecule (*CD36*), solute carrier family 2 member 4 (*GLUT4*), insulin receptor (*INSR*), peroxisome proliferator‐activated receptor gamma coactivator 1 alpha (*PPARGC1A*), perilipin 5 (*PLIN5*), peroxisome proliferator‐activated receptor alpha (*PPARA*), stearoyl‐CoA desaturase (*SCD*) and sterol regulatory element binding transcription factor 1 (*SREBF1*). *Statistically significant relative to comparative donor group (*p* < .05, unpaired Student's *t*‐test). IGR, impaired glucose regulation.

The mRNA expression of genes was measured in biopsies from the *vastus lateralis* muscle (Figure 3). There were no significant differences between control and IGR cohorts in the expression of mRNA of any of the genes studied. Muscle biopsies from athletes showed significantly higher expression, compared to both control and IGR, of the mRNAs indicating a more oxidative fiber type—*MYH7* peroxisome proliferator‐activated receptor gamma coactivator 1 alpha (*PPARGC1A*), and peroxisome proliferator‐activated receptor alpha (*PPARA*) (Figure 3A,D). In addition, tumor necrosis factor (*TNFA*) was also increased compared to control for athletes (Figure 3C).

### 3.4 Effects of DGAT inhibitors on metabolism of oleic acid in myotubes

**FIGURE 4:**
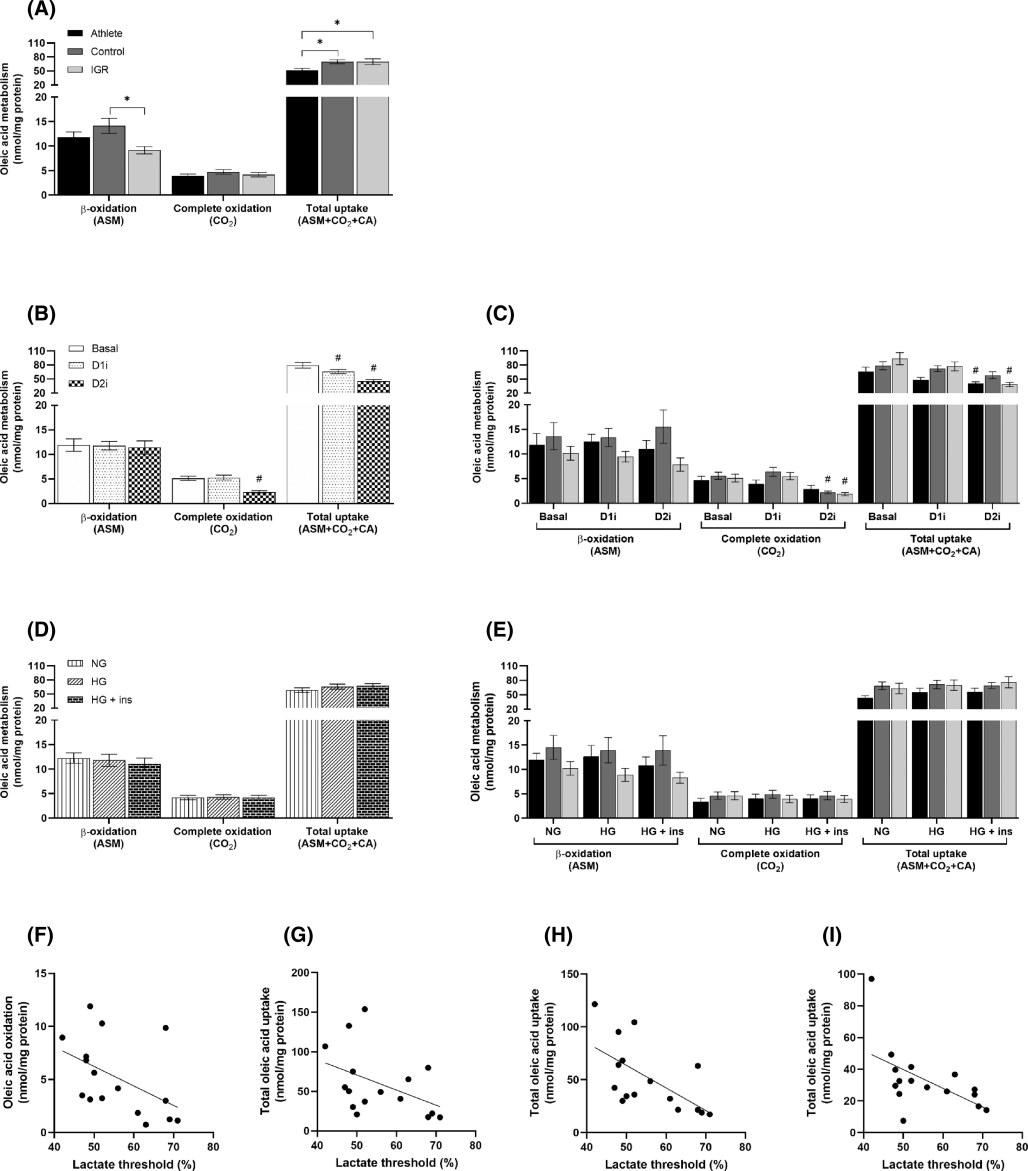
Oleic acid metabolism in myotubes derived from *vastus lateralis* muscle biopsies. Satellite cells isolated from biopsies of muscle from athletes, controls and IGR subjects were cultured and differentiated into myotubes. Cells were preincubated for 6 h in media containing either 5.5 mM or 20 mM glucose (NG and HG, respectively) in the presence or absence of 10 μM D1i or 10 μM D2i. The cells were then further incubated with 100 μM ^14^C‐oleic acid for 4 h in the presence or absence of 10 μM D1i or 10 μM D2i. Incomplete fatty acid β‐oxidation was measured as ^14^C‐acid‐soluble metabolites (ASM). Complete ^14^C‐oleic acid oxidation was measured as ^14^C‐CO_2_ and cell‐associated (CA) radioactivity. The total fatty acid uptake was calculated as the sum of ASM, CO_2_ and CA oleic acid (ASM + CO_2_ + CA). Values are presented as means ± SEM (*n* = 6 in each group) in absolute values (nmol/mg cell protein). (A) Oleic acid metabolism in myotubes from the three donor groups. (B) Effects of DGAT inhibitors on oleic acid metabolism when all myotube data is pooled. (C) Effect of DGAT inhibitors on oleic acid metabolism in cells from the three separate donor groups. (D) Pooled data for the effects of normoglycemia (NG, 5.5 mM glucose) and hyperglycemia (HG, 20 mM glucose) ± insulin (ins, 100 nM) on oleic acid metabolism. (E) Effect of NG and HG ± insulin on oleic acid metabolism in myotubes from the separate donor groups. (F–I) Correlations of individual donor anthropometric parameters with oleic acid oxidation and uptake in myotubes; Spearman's test of correlation between oleic acid oxidation and uptake in myotubes and lactate threshold of corresponding donors. The solid lines represent the regression line for all donors (*n* = 16). (F) Oleic acid oxidation in presence of D1i (*r* = −.56, *p* = .03). (G) Oleic acid uptake under NG conditions (r = −.50, *p* = .05). (H) Oleic acid uptake in presence of D1i (*r* = −.7, *p* = .002) and (I) in the presence of D2i inhibitor (*r* = −.64, *p* = .008). *Statistically significant relative to cells from comparative donor group (*p* < .05, linear mixed‐model analysis). ^#^Statistically significant relative to basal (*p* < .05, linear mixed‐model analysis). D1i, DGAT1 inhibitor (T863); D2i, DGAT2 inhibitor (JNJ‐DGAT2‐A); IGR, impaired glucose regulation.

Long‐chain FA uptake and metabolism were quantified by incubation of myotubes with ^14^C‐oleic acid (OA). Myotubes obtained from athletes had significantly lower rates of overall OA uptake compared to cells from the two other donor groups (Figure 4A). Conversely, there was no difference in total OA uptake between myotubes prepared from IGR and control groups of donors. Moreover, there were no differences between the cohorts in the proportion of OA oxidized to CO_2_, but myotubes prepared from IGR individuals had a lower proportion of incompletely oxidized OA, measured as ASMs, compared to cells from controls (Figure 4A). There were no significant correlations for OA metabolism in myotubes versus age and BMI of the donors suggesting that these anthropometric parameters did not influence the metabolic data obtained with myotubes (data not shown).

Both D1i and D2i resulted in a decrease in overall OA uptake by myotubes. This was more pronounced after DGAT2 inhibition and was statistically significant when data for all three cohorts were combined (Figure 4B). The effects of D2i on OA uptake were also significant when uptake in myotubes from IGR and athletes were plotted separately (Figure 4C). However, the mechanism for this lowering of OA uptake appeared to differ between D1i and D2i. Thus, only DGAT2 inhibition resulted in the reduction of OA oxidation to CO_2_ (Figure 4B). There were no differences between NG and HG, in either the presence or absence of insulin, on overall OA uptake or its metabolism, between donor groups (Figure 4D,E). Oxidation of OA under NG in the presence of D1i correlated negatively with the lactate threshold parameter of the donors (*r* = −.56, *p* = .03, Figure 4F). There were also negative correlations between OA uptake in myotubes kept under NG conditions and the lactate threshold of the donors (*r* = −.50, *p* = .05) (Figure 4G). Such negative correlations were also observed for NG myotubes incubated in the presence of either D1i (*r* = −.70, *p* = .002) (Figure 4H) or D2i (*r* = −.64, *p* = .008) (Figure 4I). For myotubes maintained in HG or HG + insulin we observed significant negative correlations only when myotubes were exposed to the D1i (data not shown). These observations suggest a negative relationship between the capacity for FA metabolism in muscle in vitro—especially in the presence of D1i ‐ and lactate threshold of the respective donors in vivo. This indicates that there is a strong negative correlation between the ability of muscle to oxidize pyruvate aerobically in vivo (lactate threshold) and the intrinsic capacity of the cells in vitro to take up and oxidize OA, as would be expected from the reciprocal regulation of the oxidation of pyruvate and FAs mediated at the pyruvate dehydrogenase step.

### 3.5 Effects of DGAT inhibitors on metabolism of acetate in myotubes

#### 3.5.1 Acetate uptake and oxidation

**FIGURE 5:**
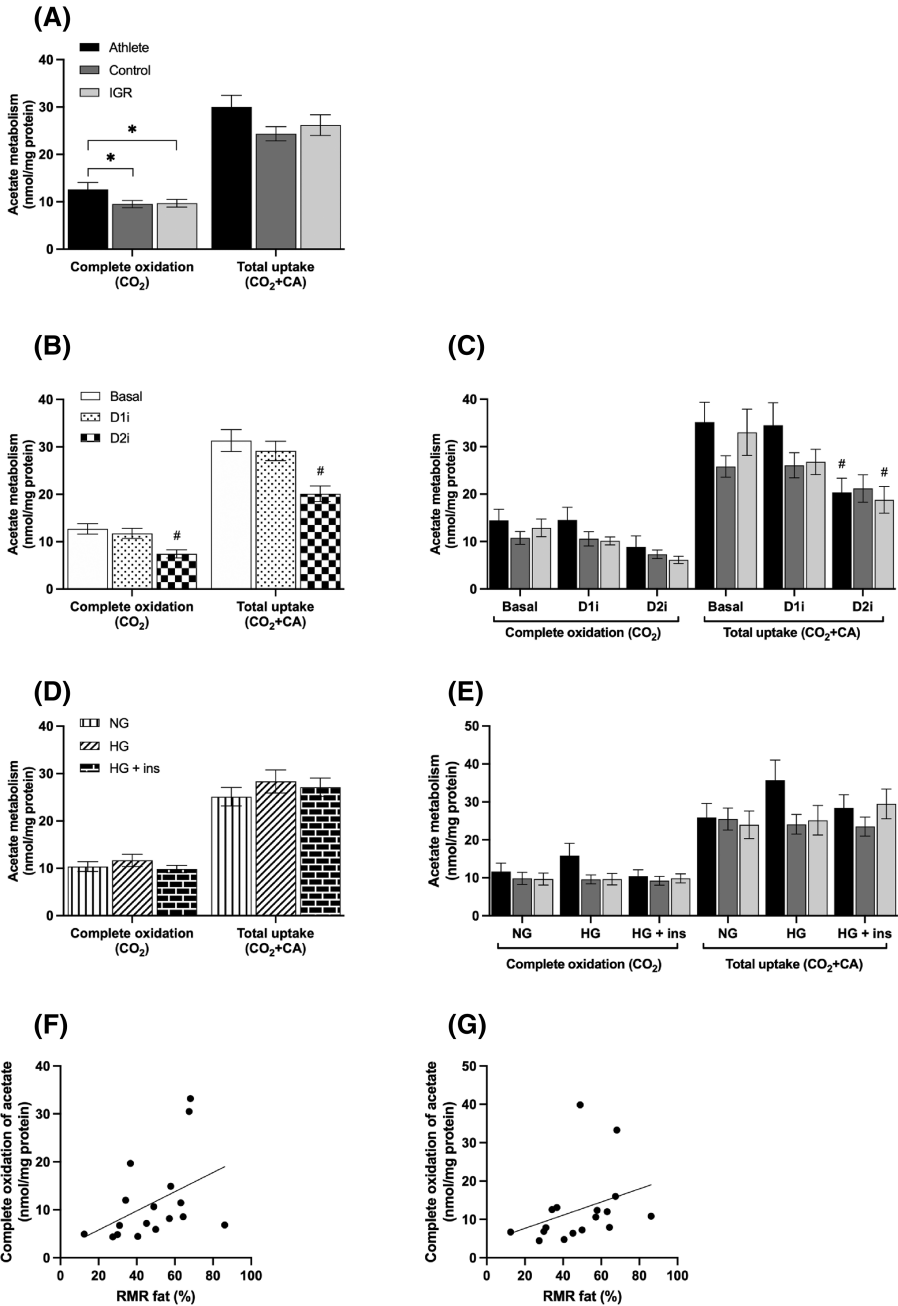
Acetate metabolism in myotubes prepared from *vastus lateralis* muscle. Satellite cells isolated from biopsies from *musculus vastus lateralis* from athletes, controls and IGR subjects were cultured and differentiated into myotubes. Cells were incubated for 6 h in media containing either 5.5 mM or 20 mM glucose (NG and HG, respectively) in the presence or absence of 10 μM D1i or 10 μM D2i. The cells were then further incubated with 100 μM ^14^C‐acetate for 4 h in the presence or absence of 10 μM D1i or 10 μM D2i. (A) Combined rates of uptake and oxidation of ^14^C‐acetate in myotubes from the three donor groups (overall effects). (B) Effects of DGAT inhibitors on acetate metabolism (overall effects on pooled data (*n* = 16) for myotubes from all three cohorts). (C) Overall effects of DGAT inhibitors on acetate metabolism in cells from all three cohorts. (D) Effect of NG and HG ± insulin (ins) on acetate metabolism (overall effects). (E) Overall effects of NG and HG ± ins on acetate metabolism in myotubes. Values are presented as means ± SEM (*n* = 6 in control group and *n* = 5 in the athletic and IGR groups, respectively) in absolute values (nmol/mg cell protein). (F and G) Correlations of donor anthropometric parameters with acetate oxidation in the presence of D1i in myotubes. Spearman's test of correlation between acetate oxidation in the presence of D1i in myotubes and RMR Fat (%) of corresponding donors. The solid lines represent the regression line for all donors (*n* = 16). (F) Acetate oxidation in the presence of D1i under NG conditions (*r* = .58, *p* = .01). (G) Acetate oxidation in the presence of D1i under HG conditions (*r* = .54, *p* = .03). *Statistically significant relative to cells from comparative donor group (*p* < .05, linear mixed‐model analysis). ^#^Statistically significant relative to basal (*p* < .05, linear mixed‐model analysis). D1i, DGAT1 inhibitor (T863); D2i, DGAT2 inhibitor (JNJ‐DGAT2‐A); HG, hyperglycemia; IGR, impaired glucose regulation; NG, normoglycemia; RMR Fat, resting metabolic rate from fatty acid oxidation in vivo.

Complete oxidation (to CO_2_) after incubation with ^14^C‐acetate was higher in cells from athletes compared to cells from control and IGR groups (Figure 5A). There were no differences between donor cohorts in uptake of acetate by myotubes (Figure 5A) and, therefore data could be combined to examine the effects of the inhibitors. This combined data (Figure 5B) showed that D2i significantly inhibited uptake as well as oxidation of ^14^C‐acetate; this reached statistical significance for uptake in myotubes derived from athletes and IGR (Figure 5C). Conversely, D1i had no effect on these parameters in myotubes from any of the cohorts. HG in the presence or absence of insulin did not affect overall ^14^C‐acetate uptake in myotubes from any of the cohorts (Figure 5D,E). Acetate oxidation in the myotubes showed a positive correlation with the proportion of whole body resting energy expenditure coming from fat oxidation (RMR Fat [%]) when myotubes were exposed to D1i under both NG (*r* = .58, *p* = .01) (Figure 5F) and HG (*r* = .54, *p* = .03) (Figure 5G) conditions. This suggests that DGAT1 inhibition resulted in greater acetate oxidation in muscle cells from subjects that had high rates of fat oxidation in vivo.

#### 3.5.2 Lipids synthesized from ^14^C‐acetate

**FIGURE 6:**
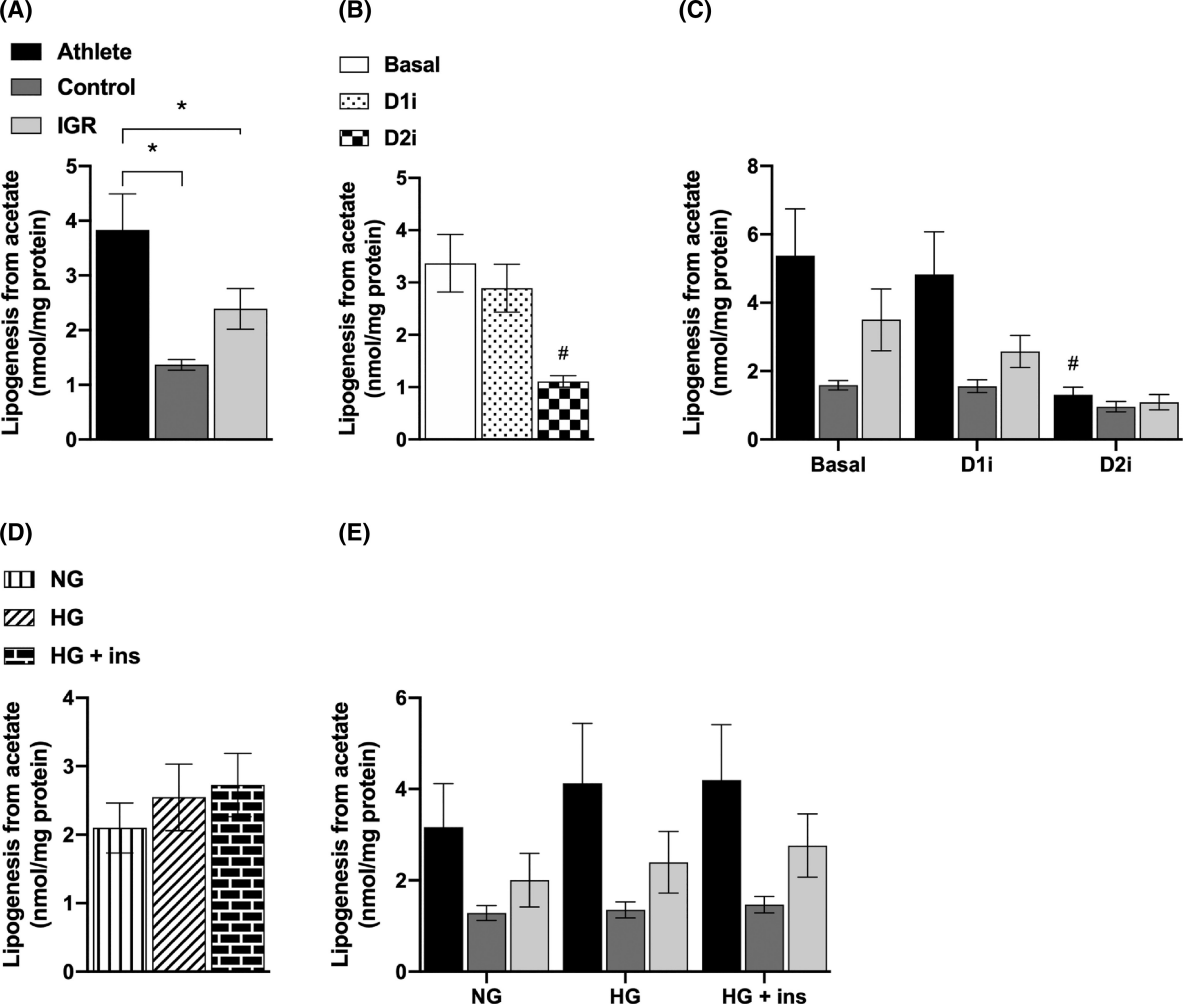
De novo lipogenesis from acetate in myotubes derived from *vastus lateralis* muscle. Satellite cells isolated from biopsies from *vastus lateralis* muscle from athletes, controls, and IGR subjects were cultured and differentiated into myotubes. Cells were incubated for 6 h in media containing either 5.5 mM or 20 mM glucose (NG and HG, respectively) in the presence or absence of 10 μM D1i or 10 μM D2i. The cells were then further incubated with 100 μM ^14^C‐acetate for 4 h under the same conditions as for the preincubation. (A) De novo lipogenesis from ^14^C‐acetate in myotubes from the three donor groups (overall effects). (B) Effects of DGAT inhibitors on de novo lipogenesis from acetate (overall effects). (C) Effect of DGAT inhibitors on de novo lipogenesis from acetate in cells from the three donor groups. (D) Effect of NG and HG ± insulin (ins) on de novo lipogenesis from acetate (overall effects). (E) Effect of NG and HG ± ins on de novo lipogenesis from acetate in cells from the three donor groups. Values are means ± SEM (*n* = 6 in control group and *n* = 5 in the athletic and IGR groups, respectively) in absolute values (nmol/mg cell protein). *Statistically significant relative to myotubes from comparative donor group (*p* < .05, linear mixed‐model analysis). ^#^Statistically significant relative to basal (*p* < .05, linear mixed‐model analysis). D1i, DGAT1 inhibitor (T863); D2i, DGAT2 inhibitor (JNJ‐DGAT2‐A); HG, hyperglycemia; IGR, impaired glucose regulation; NG, normoglycemia.

De novo lipogenesis from ^14^C‐acetate was highest in myotubes prepared from athletes (3‐fold higher than control cells); there was a smaller increase in lipogenesis in myotubes prepared from IGR compared with myotubes derived from controls (Figure 6A). When data from all cell experiments were combined, a significant inhibitory effect of D2i on lipogenesis from acetate was observed (Figure 6B). This was due to an inhibitory effect on cells from all cohorts but was especially pronounced (75% inhibition) in myotubes from athletes (Figure 6C). By contrast, DGAT1 inhibition did not affect lipogenesis from acetate in myotubes from any of the cohorts. De novo lipogenesis from acetate was not affected by HG or insulin under the conditions used (Figure 6D,E).

### 3.6 Myotube expression of mRNA for genes involved in lipogenesis and triacylglycerol synthesis

**FIGURE 7:**
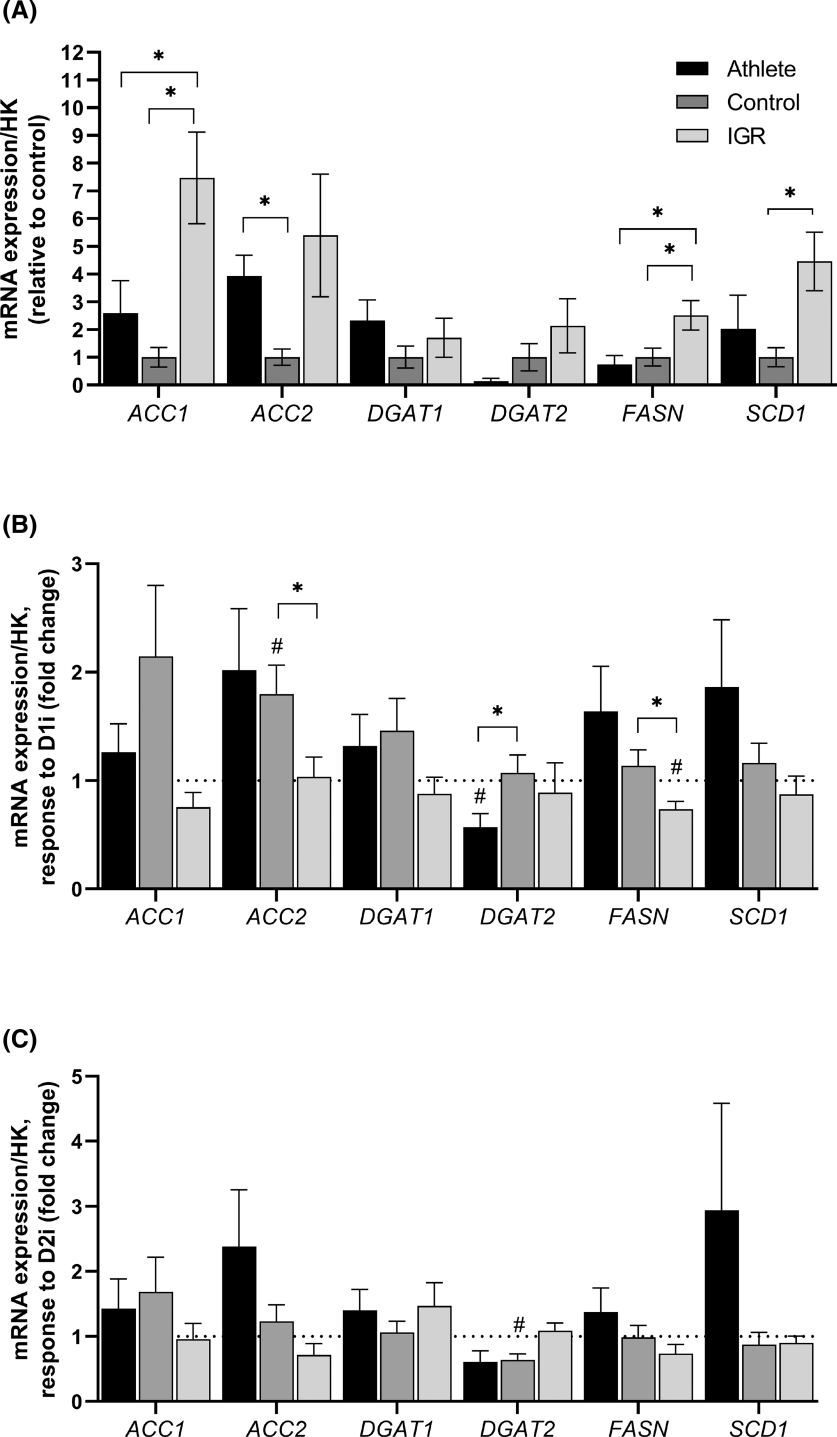
Expression of mRNA for genes of enzymes involved in lipogenesis in myotubes derived from *vastus lateralis* muscle. Satellite cells isolated from biopsies from *musculus vastus lateralis* from athletes (*n* = 7), controls (*n* = 8), and IGR (*n* = 7) subjects were cultured and differentiated into myotubes. Cells were preincubated for 6 h in media containing either 5.5 mM or 20 mM glucose (NG and HG, respectively) in the presence or absence of 10 μM D1i or 10 μM D2i before harvested for mRNA measurement. Values were normalized with respect to the expression of *L19* mRNA in each sample (HK = housekeeping). (A) mRNA expression relative to control; (B) response to D1i on mRNA expression; (C) response to D2i on mRNA expression. *ACACA* and *ACACB*, acetyl‐CoA carboxylases 1 and 2; *DGAT1* and *DGAT2*, diacylglycerol acyltransferases 1 and 2; *FASN*, fatty acid synthase; HG, hyperglycemia; IGR, impaired glucose regulation; NG, normoglycemia; *SCD1*, stearoyl‐CoA desaturase 1. *Statistically significant relative to cells from comparative donor group (*p* < .05, unpaired Student's *t*‐test).

The mRNA expression of the following genes was measured in myotubes from all three cohorts: acetyl‐CoA carboxylases 1 and 2 (*ACACA, ACACB*), *DGAT1* and *2*, stearoyl‐CoA desaturase (*SCD1*) and fatty acid synthase (*FASN*). These measurements were performed on myotubes that were either cultured under NG conditions (Figure 7) or HG conditions in the presence or absence of an maximal concentration of insulin (100 nM). The mRNA expression values obtained for myotubes incubated under NG (which were not affected by incubation with insulin) were considered to represent the basal state, which most likely reflected the phenotype of the donors (Figure 7A). These data showed that myotubes prepared from IGR individuals had higher expressions of lipogenic enzyme mRNAs (*ACACA, FASN*) compared to those from controls and athletes (for *SCD1* also higher for IGR vs. control cells). There were no changes in the mRNA expression of any of the genes studied in myotubes in response to insulin or HG. The response to DGAT inhibitors (added for 6 h prior to RNA extraction) differed between the cohorts; mRNA expression was lower for *ACACB* and *FASN* in cells from IGR subjects vs. control myotubes in response to D1i (Figure 7B); however, there were no differences between cohorts in response to D2i (Figure 7C).

### 3.7 Effects of DGAT1 and DGAT2 inhibitors on insulin‐induced Akt phosphorylation

To assess whether DGAT1 or DGAT2 inhibition influenced sensitivity of insulin signaling, we measured the Akt‐P(Ser473)/Akt ratio in myotubes incubated acutely (10 min) with 10 nM insulin. In preliminary experiments, these conditions were shown to give approximately 50% of the maximal effect of the hormone and, therefore, were suitable for determining insulin sensitivity of the activation of a downstream component of the insulin signal transduction cascade, Akt. Cells were exposed to insulin under NG or HG conditions, and in the absence or presence of D1i or D2i for 6 h before addition of insulin for 10 min. Preliminary measurements on two separate myotube preparations derived from control participants showed there were no effects of D1i or D2i on Akt‐P(Ser437) in the absence of insulin.

**FIGURE 8:**
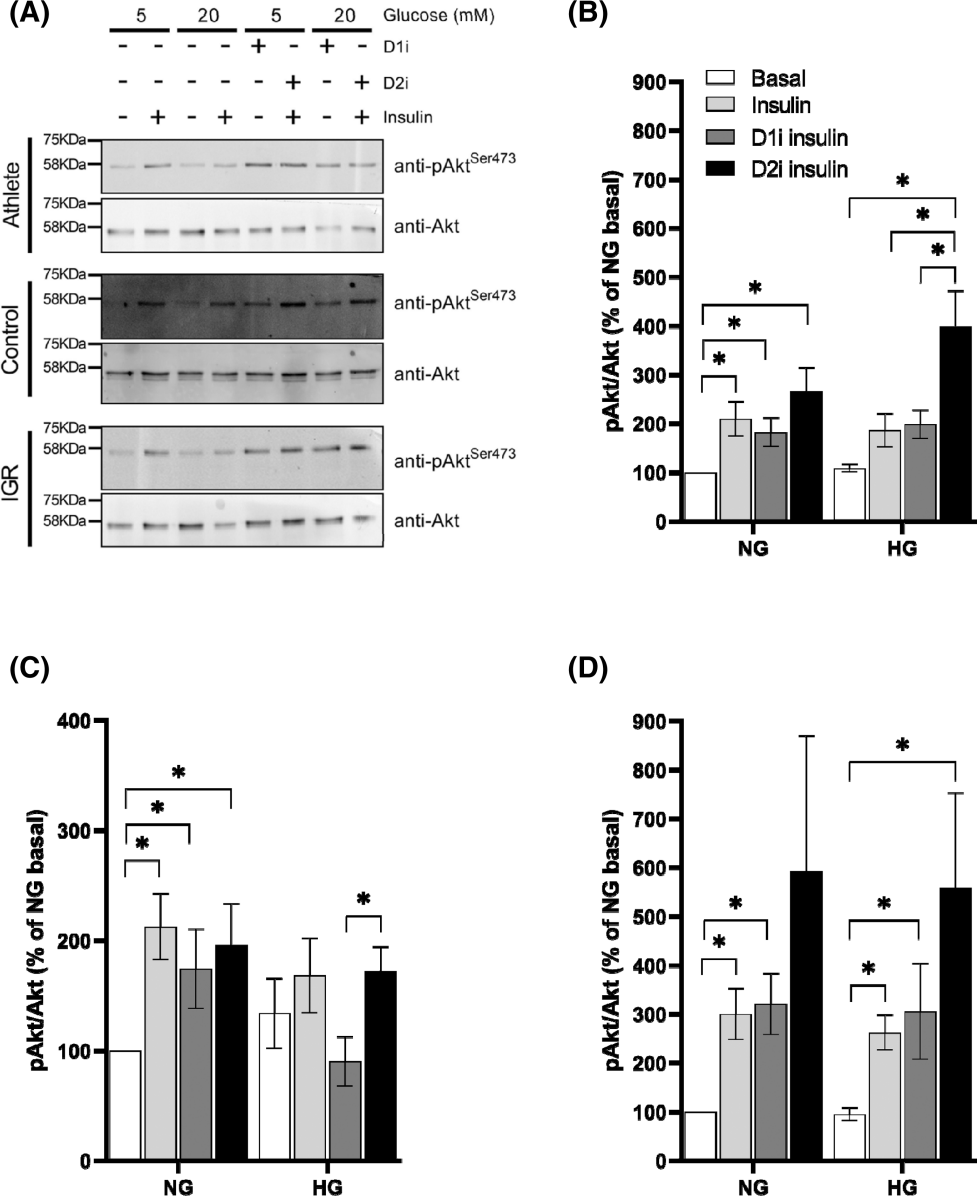
Akt phosphorylation relative to total Akt in myotubes derived from *vastus lateralis* muscle. Satellite cells isolated from biopsies from *vastus lateralis* muscle from athletes, controls and IGR individuals were cultured and differentiated into myotubes. Cells were incubated for 6 h in media containing either 5.5 mM or 20 mM glucose (NG and HG, respectively) in the presence or absence of 10 μM D1i or 10 μM D2i, followed by incubation in the presence or absence of 10 nM insulin for 10 min. Lysates were prepared and lysate proteins resolved by SDS‐PAGE prior to immunoblotting with anti‐Akt or anti‐phospho‐Akt(Ser473) antibodies. Representative blots for each cohort obtained using the LICOR method are given in (A). The bands were quantified and the ratio Akt‐P(Ser473)/Akt total for each incubation condition within the same experiment was computed. Values for the fold‐increase in this ratio relative to that for cells incubated under NG in the same preparation are presented. Values are means ± SEM for myotubes derived from (B) Control (*n* = 8), (C) Athlete (*n* = 6) and (D) IGR (*n* = 6) donors. Asterisks denote statistical significance of difference (*p* < .05) between values for the indicated incubation conditions. NG (5.5 mM glucose); HG (20.0 mM glucose); D1i, DGAT1 inhibitor; D2i, DGAT2 inhibitor.; insulin = 10 nM for 10 min.

The analysis of the Akt phosphorylation data is limited by the fact that only the increase in phosphorylation of the Ser473 site was measured after stimulation by insulin at a sub‐maximal concentration (10 nM). However, potentially important trends were observed. Thus, under NG conditions, the fold increase in Akt‐P/total Akt ratio mediated by insulin was similar for cells from all three cohorts. Moreover, cells derived from the control and IGR cohorts gave a very similar pattern of responses under both NG and HG conditions (Figure 8B,D). Specifically, (i) DGAT1 inhibition had no effect on the insulin‐mediated increase in Akt‐P/Akt ratio; (ii) DGAT2 inhibition tended to increase insulin‐mediated Akt phosphorylation especially under HG conditions. This pattern resulted in consistently higher Akt‐P responses after incubation of cells with DGAT2i compared to DGAT1i treatment. The difference reached statistical significance in cells derived from the control cohort maintained under HG conditions (Figure 8B). These observations indicate that DGAT2 inhibition increased the sensitivity of the Akt‐P response to insulin in cells from control and IGR cohorts.

Cells derived from athletes showed several differences from the above pattern of responses (Figure 8C). In particular, their basal (no insulin) Akt‐P/Akt ratio was raised under HG conditions such that there was no further significant increase of Akt‐P/Akt induced by insulin or by DGAT2 inhibition at 20 mM glucose (Figure 8C). This was in marked contrast to the results observed for myotubes from control and IGR individuals. Moreover, in athlete‐derived myotubes, DGAT1 inhibition attenuated the increase in Akt‐P/Akt response to insulin under HG conditions. Consequently, the significant difference between Akt‐P/Akt ratio between cells treated with D1i or D2i (observed for control‐derived cells) was maintained in IGR‐derived cells.

The above observations suggest that DGAT1 and DGAT2 inhibitions have opposing effects on Akt‐P responses to insulin particularly under hyperglycemic conditions.

## 4 DISCUSSION

The primary aim of this study was to ascertain whether DGAT1 and DGAT2 have distinctive roles in skeletal muscle FA metabolism—as previously demonstrated in other tissues—and whether this difference is reflected in myotubes prepared from human cohorts that had previously been shown to have similar IMTG content but differing in insulin sensitivity. In addition, we wanted to examine whether the route of TAG synthesis (via DGAT1 or DGAT2) affected insulin signaling in muscle cells since TAG hydrolysis is known to generate metabolites that affect insulin signaling and FA oxidation. These possibilities arose from the known specialization of DGAT2 activity to synthesize TAG from de novo synthesized FA and nascent DAG, which is anticipated to result in TAG of distinctive composition and LD localization, which upon lipolysis gives rise to activators of transcription factors that may affect metabolism more widely.^19^, ^20^, ^36^, ^37^ We examined whether the route through which TAG is synthesized in myotubes (i.e., via DGAT1 or DGAT2) affected FA metabolism sufficiently to modulate insulin signaling in myotubes derived from athletes or individuals with IGR, as these two phenotypes are known to have similar levels of IMTG but widely different insulin sensitivities—reviewed in Ref.^5^ Therefore, we determined several physiological characteristics of individuals recruited to the three cohorts. These can be summarized as showing that IGR individuals had significantly higher HOMA‐IR values, lower markers of insulin sensitivity, and higher HbA1c values. The athlete cohort, while having higher VO_2max_ levels, did not have significantly higher ISI or lower BMI than control subjects.

Measurement of a panel of mRNAs in biopsies from *vastus lateralis* muscle of genes involved in lipid metabolism indicated that there were no differences in mRNA expression between controls and IGR. But athletes, compared to the other cohorts, showed significantly higher mRNA expression of *PPARA*, a transcription factor that promotes mitochondrial function, reflecting a higher importance of FA metabolism in the muscle of these participants.

We also characterized the LD numbers per unit cell area in type I and type II muscle fibers in cryosections obtained from biopsy samples. The method used for quantifying LD numbers and sizes did not enable us to quantify intracellular lipid mass (volume) but we could compute the cross‐sectional area of LDs expressed per unit cell area (LD‐CSA). The higher LD‐CSA in type I fibers (significant in athletes compared to controls) was as expected from their higher oxidative capacity and was associated primarily with a higher proportion of lipid contained in larger LDs. Remarkably, the LD‐CSA of type I and type II fibers was very similar in muscles from IGR subjects due to a significantly higher LD content of type II fibers in IGR compared to either control or athlete biopsies. Therefore, the increased IMTG of skeletal muscle previously observed in vivo in IGR subjects^1^, ^2^ appears to be due primarily to an increase of LD number per unit area in type II fibers. This was consistent with the observation that, in myotubes cultured under NG conditions, IGR‐derived myotubes were the only ones to express higher levels of lipogenic enzyme mRNAs (*ACACA*, *SCD1*, and *FASN*) compared to those from controls, indicating that the phenotypic differences in donors are retained in myotubes. This suggests that de novo FA synthesis may play an enhanced role in determining the lipid content of type II fibers in skeletal muscle of IGR individuals. Although no statistically significant differences were found, this is also consistent with the observation that *DGAT2* mRNA expression tended to be higher in myotubes derived from IGR individuals relative to controls whereas this did not occur in myotubes from athletes. This possible contrasting pattern of *DGAT*2 mRNA expression in myotubes derived from IGR and athlete cohorts could be important when considered alongside the increase in LD‐CSA of type II fibers in sections of biopsies obtained from IGR subjects. It suggests that the well‐established increases in IMTG of skeletal muscle in IGR and athletes^3^ may occur differently, namely, primarily in type II fibers in IGR and in and type I fibers in athletes. This divergence may contribute towards the apparent “paradox” of equally high overall IMTG but divergent muscle insulin sensitivity in these two physiological conditions.^5^

The role that DGAT2 may play in this divergence between the two cohorts was apparent when myotubes were incubated with acetate as a lipogenic substrate. Inhibition of DGAT2 resulted in a 30% lowering of ^14^C‐acetate incorporation into cell‐associated lipids. Conversely, there was no such effect when DGAT1 was inhibited. These data confirm that, as in other cell‐types, DGAT2 is specialized for the synthesis of TAG from de novo synthesized FAs in skeletal muscle.

Interestingly, DGAT2 inhibition also appeared to play a role in determining mitochondrial long‐chain FA oxidation. The combined data for all myotubes incubated with D1i and OA showed that, at the OA concentrations used, there was no significant competition for intracellular oleoyl‐CoA between FA oxidation and glyceride synthesis, as D1i had no effect on the uptake or oxidation of OA. By contrast, myotubes incubated with D2i showed the lowest rate of OA oxidation, which was accompanied by a lower overall OA uptake. This is the inverse of what would be expected if inhibition of DGAT2‐mediated TAG synthesis was sparing oleoyl‐CoA for oxidation. Consequently, this effect of D2i is unlikely to have been dependent on substrate‐level changes in the competition between FA oxidation and TAG synthesis, suggesting that inhibition of DGAT2‐mediated TAG synthesis impairs a process that promotes mitochondrial FA oxidation. TAG are known to be the source of lipolytic products that serve as modulators for transcription factors.^19^, ^21^, ^37^ Therefore, the data indicate that DGAT2‐derived TAG act as sources of metabolites that activate FA oxidation in skeletal muscle cells. This suggests that although expression of DGAT2 in skeletal muscle is low in absolute terms and its activity is unlikely to compete quantitatively with FA oxidation for acyl‐CoA substrate, DGAT2 activity is indirectly involved in the maintenance of mitochondrial metabolism of FA.

We hypothesized that this specialized role of DGAT2 may extend to the formation of lipid effectors (TAG hydrolysis products) that modulate insulin signaling, in view of previous studies that showed that overexpression of DGAT2 in type II fibers in mice resulted in decreased insulin sensitivity.^26^ This was tested directly by studying the effects of the inhibition of either DGAT1 or DGAT2 in myotubes on Akt phosphorylation (Akt‐P(Ser473)/total Akt ratio) upon acute exposure of myotubes to an approximately half‐maximally effective concentration of insulin (10 nM). Because we only measured phosphorylation on a single site in Akt the inferences that could be drawn about “insulin signaling” are necessarily limited. In myotubes derived from all three cohorts, Akt‐P/Akt was highest after D2i treatment although this reached statistical significance only in myotubes obtained from controls, and maintained under HG, conditions that are known to increase rates of de novo FA synthesis in myotubes.^23^ Moreover, in the cells derived from athletes, in which Akt phosphorylation was enhanced under basal HG conditions, DGAT1 inhibition attenuated the effect of insulin on Akt phosphorylation, resulting in a highly statistically significant difference between Akt‐P/Akt ratios in cells treated with D1i or D2i. This suggests that inhibition of DGAT1 and DGAT2 has opposite effects on insulin sensitivity in myotubes from athletes, with DGAT1 activity enhancing insulin sensitivity while DGAT2 activity decreasing insulin sensitivity. These inferences are consistent with previous conclusions obtained after overexpression of DGAT1 in cardiac and skeletal muscle^24^, ^25^ or DGAT2 in type II skeletal muscle fibers^25^ in mice in vivo.

**FIGURE 9:**
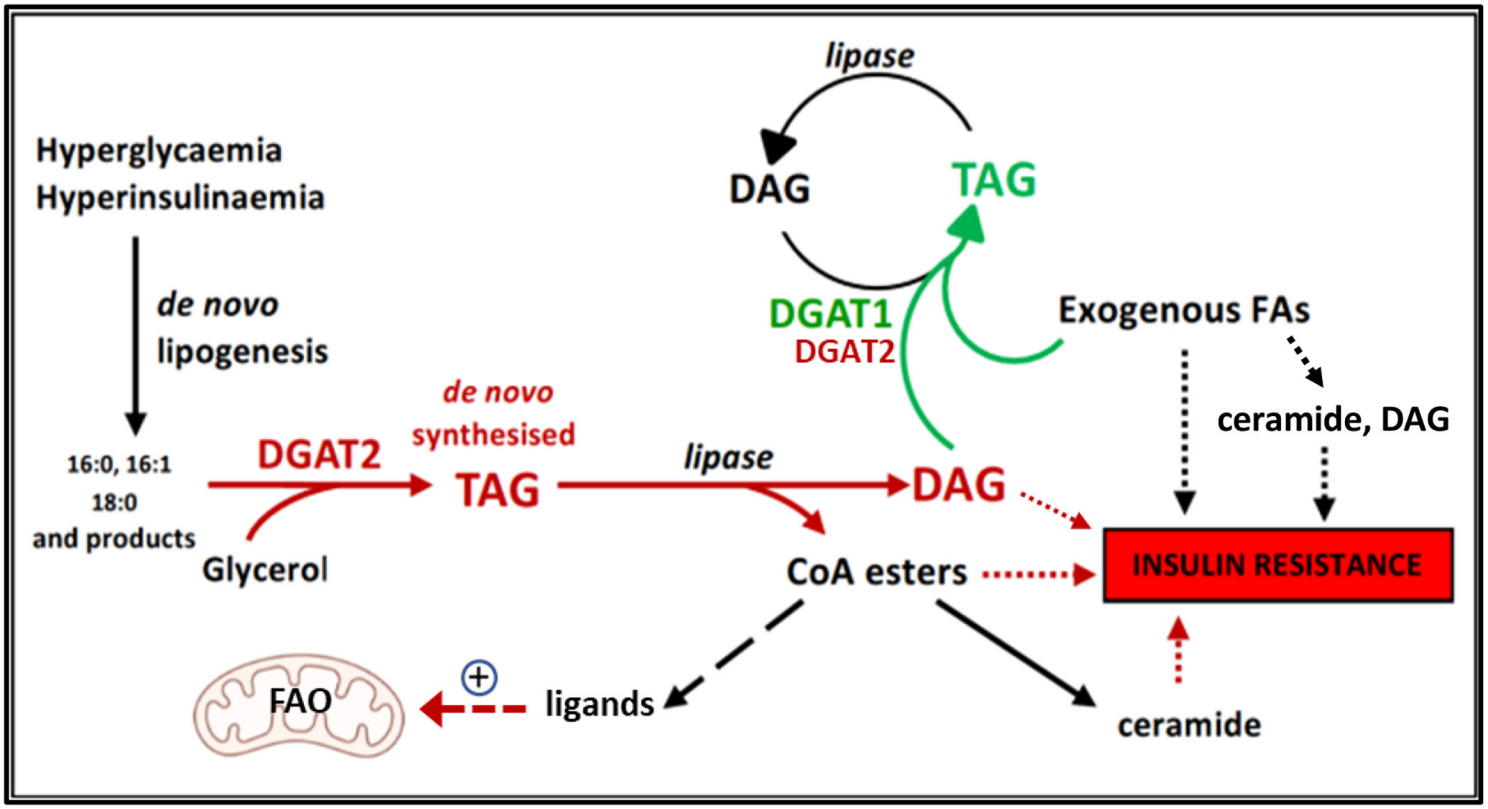
Schematic representation of the proposed role of DGAT1 and DGAT2 in generating different pools of TAG. DGAT2 participates in TAG synthesis from both preformed FA and from de novo synthesized FA, whereas DGAT1 only uses preformed FA. The composition of TAG synthesized by DGAT2 from nascent DAG and de novo FA is distinct and, upon hydrolysis, generates lipid metabolites that affect insulin signaling and mitochondrial function. DGAT1 and 2, diacylglycerol acyltransferases 1 and 2; FA, fatty acid; FAO, fatty acid oxidation; TAG, triacylglycerol.

This concept and other inferences from this study are illustrated graphically in Figure 9, which shows the current state of knowledge of the relationship between the respective roles of DGAT1 and DGAT2. According to this construct, whereas DGAT1 plays an insulin‐sensitizing role by directly or indirectly sequestering molecular species that promote insulin resistance, DGAT2 has the capacity to utilize de novo synthesized FA and nascent DAG as substrates to form a distinct pool of TAG which, upon hydrolysis, generates lipid metabolites that affect insulin signaling. Additional findings in this study suggest that such a signaling function of DGAT2 may extend to the regulation of mitochondrial FA oxidation.

There were no significant correlations for OA metabolism in myotubes with age and BMI of the donors suggesting that these anthropometric parameters did not influence the metabolic data obtained using myotubes. However, we found interesting correlations between some physiological parameters of the donors and some metabolic characteristics of myotubes prepared from their muscle biopsies. In particular, we found a strong negative correlation between OA uptake and oxidation in myotubes and the lactate thresholds of the respective donor individuals. This observation indicates that there is a strong inverse relationship between the ability of muscle to aerobically oxidize glycolytically generated pyruvate in vivo (lactate threshold) and the uptake and oxidation of OA in myotubes in vitro as would be expected from the reciprocal regulation of mitochondrial oxidation of pyruvate and FAs mediated at the pyruvate dehydrogenase step. This provides further evidence that myotubes retain the phenotypic properties of the cognate donors.

Following the highlighting of the pivotal role of DGAT2 in hepatocytes where it is suggested to act as the link between glycemia, steatosis, and dyslipidemia^15^, ^38^ and, soon afterwards, of the unsuccessful phase 1 trials of DGAT1 inhibitors in humans,^39^ the interest in pharmacological manipulation of DGAT activities has shifted from DGAT1^40^ to DGAT2.^41^ The present results indicate that a future pharmacological strategy for the development of the use of DGAT2 inhibitors in humans may benefit from the present observations, namely, the increased insulin signaling and the lowering of long‐chain FA oxidation that DGAT2 inhibition may induce in skeletal muscle in addition to its roles in the liver.^38^, ^41^

## AUTHOR CONTRIBUTIONS

Zehra Irshad, Jenny Lund, Nils Gunnar Løvsletten, Anne Sillars, Seley Gharanei performed experiments, collated data, and wrote parts of the manuscript. Jenny Lund performed the statistical analyses. Victor A. Zammit, Dilys J. Freeman, Jason M. R. Gill, Ian P. Salt, Arild C. Rustan, G. Hege Thoresen planned experiments, interpreted the data, and wrote the manuscript.

## DISCLOSURES

There are no conflicts of interests to declare by any of the authors.

## Supporting information


Data S1

## Data Availability

There are no missing data relevant to the manuscript. All the data and methods are included within the manuscript and Supplementary data provided.
