# Supplementary material for: The roles of DGAT1 and DGAT2 in human myotubes are dependent on donor patho‐physiological background
Source: FASEB J. 2023 Oct 1;37(11):e23209. doi: 10.1096/fj.202300960RR (PMC10947296; doi:10.1096/fj.202300960RR)
Supplement: Supplementary file 1 — Data S1 [file FSB2-37-0-s001.docx]

**Data S1**

Primer sequences for quantification of selected gene mRNA in myotubes

| **Gene** | **Forward** | **Reverse** |
| --- | --- | --- |
|  |  |  |
| hACC1 | 5 ′-TTTGACCTCACTGCCATTCC-3 ′ | 5 ′-GCGACTTCCATACCGCATTAC-3 ′ |
| hACC2 | 5'-CAAGTGCAAGATCTGTTTCCCTGATCG-3' | 5'-CCGGAAGTTTAGGGTTTTCTGAAGCAT-3' |
| hFASN | 5 ′-CGCGTGGCCGGCTACTCCTAC-3 ′ | 5 ′-CGGCTGCCACACGCTCCTCT-3 ′ |
| hSCD1 | 5 ′-TGGTGATGTTCCAGAGGAGGTA-3 ′ | 5 ′-AATGTGGTGAAGTTGATGTGCC-3 ′ |
| hDGAT1 | 5'-ACCTCATCTGGCTCATCTTCTTC-3' | 5'-GAACTCCGGTCTCCAAACTG-3' |
| hDGAT2 | 5'-GCACAGAGGCCACAGAAGTG-3' | 5'-CCCTCAACACAGGCATTCG-3' |
| hL19 | 5'-GCGGAAGGGTACAGCCAA-3' | 5'-GCAGCCGGCGCAAAA-3' |
